# Molecular characterization and pathogenicity study of a highly pathogenic strain of chicken anemia virus that emerged in China

**DOI:** 10.3389/fcimb.2023.1171622

**Published:** 2023-05-22

**Authors:** Lichun Fang, Huiyue Jia, Yuhang Hu, Yixin Wang, Zhizhong Cui, Lihong Qi, Peng Zhao

**Affiliations:** ^1^ Innovation Team for Major Livestock and Poultry Disease Prevention and Control, Shandong Academy of Agricultural Sciences, Jinan, Shandong, China; ^2^ College of Animal Science and Veterinary Medicine, Shandong Agricultural University, Tai’an, Shandong, China; ^3^ College of Animal Science and Veterinary Medicine, Henan Institute of Science and Technology, Xinxiang, Henan, China

**Keywords:** chicken anemia virus, immunosuppressive virus, severe anemia, molecular characteristics, pathogenicity

## Abstract

Chicken infectious anemia (CIA) is caused by chicken anemia virus (CAV). Recently, severe anemia has emerged in layer chickens (8 to 10-week-old) on poultry farms in China. However, the etiological characteristics and pathogenic potential of CAV in chickens at 6 weeks or older are not well understood. In this study, we isolated a CAV strain, termed SD15, from two-month-old chicken with severe anemia and analyzed the genetic evolution relationship. We found that strain SD15 had the highest homology (98.9%) with CAV18 strain. Comparison with 33 reference strains revealed 16 amino acid mutations in strain SD15, two of which were previously unknown (F210S in VP1 and L25S in Vp3). Compared with low pathogenic strains (Cux-1 and C14), highly pathogenic strains (SDLY08 and SD15) had three base mutations in their noncoding region. To further understand its pathogenicity, 10-week-old specific-pathogen-free (SPF) chickens were challenged with the novel strain and SDLY08. No obvious clinical symptoms were observed in the SDLY08 group. However, SD15-infected chickens showed significant growth retardation and immunosuppression. The main manifestations of immunosuppression were the significantly reduced thymus and bursa indices and AIV-H9 vaccine-induced antibody levels (P < 0.05). The lowest number of red blood cells in the SD15 group was just 60% of that in the control group. Taken together, the novel strain SD15 not only showed higher pathogenicity but also exhibited the potential ability to break the age resistance of older chickens to CAV. Our study enhanced the understanding of the epidemiological characteristics of chickens infected with severe anemia and can facilitate the development of improved control strategies of CIA in China.

## 1 Introduction

Chicken infectious anemia (CIA) is an immunosuppressive disease caused by chicken anemia virus (CAV), which invades bone marrow hematopoietic cells and T lymphocytoblasts of immune organs (e.g., thymus) in young chickens, causing severe anemia and immunosuppression, leading to increased mortality due to secondary complications. CAV spreads both horizontally and vertically (Jørgensen et al., 1995; Todd, 2000). It was first isolated in Japan in 1979 (Yuasa et al., 1979) and has been commonly identified since then in chicken populations worldwide (Rosenberger and Cloud, 1989; McNully, 1991; Zhou et al., 1996; Natesan et al., 2006; Craig et al., 2009; Kim et al., 2010; Zhang et al., 2013).

CAV is a member of the genus *Gyrovirus* that belongs to the family Circoviridae, and is characterized by its spherical shape, single-stranded circular DNA structure, and lack of capsule (Ducatez et al., 2005; Natesan et al., 2006). The genome of CAV is 2298–2319 nucleotides in length and comprises three major partially overlapping open reading frames that encode peptides of 51.6 (VP1), 24 (VP2), and 13.6 (VP3) kDa (Noteborn et al., 1991). The noncoding region of the CAV genome is only 0.3 kb, but shows complete promoter activity, containing more than a dozen conserved sequences related to replication and transcriptional regulation (Meehan et al., 1992). According to previous studies, the genome and amino acid composition of CAV are highly conserved, with only minor variations in predicted amino acid sequences being detected in isolates from different parts of the world. However, the pathogenicity of different strains varies greatly. Yamaguchi et al. reported that even a single change in the residue 394 of VP1 was crucial for the pathogenicity of CAV (Yamaguchi et al., 2001). Likewise, Renshaw et al. reported that amino acids 139 and 144 play vital roles in viral growth and spread in culture (Renshaw et al., 1996).

The pathologic effect of CAV is most likely to be observed in 2 to 4-week-old broilers and layers (Bülow and Sebat, 1997). The development of age-related resistance against CAV is closely related to maturation and a developed immune system, which is complete by the age of 3 weeks or even earlier (Yuasa et al., 1979; Yuasa and Imai, 1986). The degree of resistance might vary depending on the virulence of the virus, dose, and route of infection (Naish, 1989). In fact, chickens are susceptible to infection at all ages, with older affected chickens showing subclinical symptoms of immunosuppression and being more sensitive to other infections, exhibiting poor vaccine responses (van Santen et al., 2001). In Chile, CAV was isolated for the first time from thymus samples of 9-week-old White Leghorn breeders (Toro et al., 1994). Despite no antigenic differences identified between different CAV isolates in terms of cross-neutralization and cross-immunofluorescence tests, the incidence of anemia induced by different isolates of CAV was reported to have ranged between 0 and 88%, indicating differences in the virulence of different CAV strains (Yuasa et al., 1979; Bülow et al., 1986).

In China, CAV was first isolated in the Heilongjiang Province in 1992 (Cui et al., 1992). Commercialized vaccines are banned in China as they have been suggested to cause subclinical symptoms. Owing to this, CAV infections are prevalent in China, with the rate of natural infections being as high as 40–70% and seropositivity rate even reaching 100% in commercial chicken farms. Li et al. have reported that the CAV detection positive rate of chicken samples from 13 provinces of China was 14.43%, and the positive flock rate was 22.31% (Li et al., 2021). Few studies have explored the effects of CAV infection in chickens older than 4 weeks and none performed any genetic characterization (Yuasa et al., 1983; Joiner et al., 2005; Haridy et al., 2012). At present, the pathogenicity of CAV in older chickens is poorly understood.

In this study, we first report the isolation of a highly pathogenic CAV strain in older chickens (2-month-old chickens) in China. The aim of this study was to observe the molecular biological characteristics of this highly pathogenic strain not only according to the changes in the amino acid sequence of the coding region, but also those in non-coding regions. In addition, we performed viral pathogenicity tests both *in vitro* (MSB1 cells) and *in vivo* (10-week-old SPF chickens).

## 2 Materials and methods

### 2.1 Samples background

Recently, there have been many outbreaks of CAV infections in many provinces of China (Shandong, Henan, Jiangsu, etc). In this study, a CAV strain was isolated from two-month-old chickens in a large-scale layer farm in the Shandong Province, China. This was the first time that severe anemia was observed in chickens older than 8 weeks of age in China. The major clinical manifestation was the pale comb of chicken. The main feature detected in pathological examination was the pink or even pale yellow color of the bone marrow.

### 2.2 Isolation and titration of virus

Detection of CAV in bone marrow samples was performed using a PCR assay with the following primers: forward: 5′-GCATTCCGAGTGGTTACTATTCC-3′; reverse: 5′-CGTCTTGC CATCTTACAGTCTTAT-3′. All samples were also strictly tested for other viruses, such as avian leukosis virus (ALV), avian influenza virus (AIV), Newcastle virus (NDV), reticuloendotheliosis virus (REV), and fowl adenovirus (FAdV) according to published methods (Fang et al., 2017; Meng et al., 2018).

Isolation of CAV was attempted from bone marrow samples that were positive for CAV only. PCR positive samples were propagated in Marek’s disease virus transformed MDCC-MSB1 cells (American Type Culture Collection), as previously described (Goryo et al., 1987). First, 0.5 mL of the filtered sample supernatant was mixed with the MSB1 cell pellet, and then re-suspended in 0.5 mL of RPMI 1640 medium (Sigma, St. Louis, MO, USA) and incubated at 37°C for 1 h. Then, 5 mL RPMI 1640 medium was added to the mixture, and cells were incubated at 37 °C until the appearance of cytopathogenic effects (CPE). Blind passaging was carried out every 3 d. Following this, the media was collected and repeatedly frozen and thawed thrice. Finally, the presence of virus was detected by PCR. The supernatants were stored at -80°C and used for CAV propagation.

Viral titer was measured using 50% egg infectious dose (EID_50_) assays in embryonating chicken eggs (ECE), as previously described (Senne, 2019).

### 2.3 Proliferation of CAV cultured in MDCC-MSB1 cells

The isolated CAV strain was propagated on MDCC-MSB1 cells in a 6-well plate. Immunological fluorescence assay (IFA) was performed to observe the number of cells infected with CAV at 12, 36, and 48 h after infection. Cells were fixed with 4% paraformaldehyde in phosphate-buffered saline (PBS) for 20 min at 25°C, and IFA was performed using mouse anti-CAV-VP3 monospecific serum (prepared in our laboratory). Then, MSB1 cells were stained with fluorescein-isothiocyanate-labelled goat-anti-mouse antibodies (Sigma, St. Louis, MO, USA).

### 2.4 Amplification, cloning, and sequencing of the CAV genome

Based on the CAV sequences published in GenBank (**Table 1**), three pairs of primers were designed and synthesized using DNA Star 6.0 (**Table 2**). Total DNA was extracted from positive bone marrow samples and cultured cell supernatants. The amplified fragments were 843, 989, and 802 bp in length, covering the entire CAV genome. PCR amplification was performed in a 50 μL volume containing 25 μL buffer I, 16 μL dNTPs, 0.5 μL of each primer, 13.5 μL distilled water, 1 μL of DNA, and 0.5 μL LA Taq polymerase (TaKaRa, Biotechnology, Dalian, China). All PCR amplification products were analyzed by agarose gel electrophoresis followed by staining with ethidium bromide. PCR products were purified using a Gel Band Purification Kit (Omega Bio-Tek, USA), cloned into the pMD18-T vector (TaKaRa Bio Inc., Japan), and sequenced in triplicate using an ABI 3730 Sanger-based genetic analyzer (Applied Biosystems, Carlsbad, CA, USA).

**Table 1:** The GenBank accession numbers of full-length CAV genomes.

Accession number	Strain name	Host	Year	Country (area)	Whole length
M55918	Cux-1	Chicken	1991	USA	2319 bp
NC001427	NC001427	Chicken	1992	USA	2319 bp
M81223	M81223	Chicken	1993	Germany	2298 bp
CAU65414	CAU65414	Chicken	1996	Australia	2298 bp
CAU66304	CAU66304	Chicken	1997	UK	2319 bp
AB027470	TR20	Chicken	1999	Japan	2298 bp
AB031296	A2	Chicken	2000	Japan	2298 bp
AF313470	AF313470	Chicken	2000	USA	2294 bp
AF227982	AF227982	Chicken	2001	Australia	2286 bp
AB046590	AB046590	Chicken	2001	Japan	2298 bp
AF475908	AF475908	Chicken	2002	China	2298 bp
AJ297685	clone 34	Chicken	2002	Germany	2297 bp
AF390102	SMSC-1P60	Chicken	2003	Malaysia	2298 bp
AF285882	SMSC-1	Chicken	2003	Malaysia	2298 bp
AF395114	BD-3	Chicken	2004	Bangladesh	2298 bp
EF176599	C14	Chicken	2004	China	2298bp
DQ141673	SD22	Chicken	2005	China	2298 bp
DQ217401	SMSC-1P123WT	Chicken	2005	Malaysia	2298 bp
D10068	CAE26P4	Chicken	2007	Netherlands	2298 bp
EF683159	3711	Chicken	2007	Australia	2279 bp
DQ991394	01-4201	Chicken	2007	USA	2298 bp
FJ172347	SDLY08	Chicken	2008	China	2298 bp
D31965	CAECA123	Chicken	2008	Japan	2319 bp
AB119448	AB119448	Chicken	2009	Japan	2298 bp
AF311892	98D02152	Chicken	2010	USA	2298 bp
JX260426	GD-1-12	Chicken	2012	China	2298 bp
JX964755	GXC060821	Chicken	2012	China	2292 bp
JQ690762	AGV2	Human	2012	China	2316 bp
KF224935	GD-K-12	Chicken	2013	China	2298 bp
KJ872513	CIAV-10	Chicken	2014	Argentina	2298 bp
KM496307	SC-MZ42A	Chicken	2014	China	2298 bp
KC414026	CAT-Gyv	Cat	2014	China	2295 bp
KJ728827	Isolate 18	Chicken	2014	Taiwan	2298bp
KX811526	SD15	Chicken	2015	China	2298bp

**Table 2:** Primers used for genome amplification.

Primers	Sequence	Product length
F1	5’- GCATTCCGAGTGGTTACTATTCC-3’	843bp
R1	5’- CGTCTTGCCATCTTACAGTCTTAT-3’	
F2	5’- CGAGTACAGGGTAAGCGAGCTAAA-3’	989bp
R2	5’- TGCTATTCATGCAGCGGACTT-3’	
F3	5’- ACGAGCAACAGTACCCTGCTAT-3’	802bp
R3	5’- CTGTACATGCTCCACTCGTT-3’	

### 2.5 Whole genome sequence alignment and phylogenetic analysis

To establish the genotypes and clusters of the sequenced CAV strain of this study, a phylogenetic analysis based on the complete genome was performed. The complete nucleotide sequence of the novel CAV strain and reference sequences were available from GenBank (**Table 1**). Both DNA and amino acid sequences (VP1, VP2, and VP3) were assembled using DNAStar (version 7; Madison, WI, USA). Multiple-sequence alignment was performed using Clustal W (BioEdit version 7). A neighbor joining (NJ) tree based on the full-length nucleic acid sequence was constructed using the MEGA 5.1 program (Tamura et al., 2011). The robustness of the NJ tree was evaluated by bootstrap analysis with 1000 replicates.

### 2.6 Animal experimental design

Sixty 10-week-old SPF chickens (SPAFAS poultry company, Jinan, China) were randomly divided into three groups. Each group had 20 chickens, which were separately bred in shielded cages with positive filtered air. Twenty chickens in the SD15 group were challenged with the novel strain SD15 (a dose of 1500 EID_50_) *via* intramuscular injection. To better elucidate the pathogenicity of the novel strain, another infection group (20 chickens challenged with the highly pathogenic strain SDLY08 at the same dose) was added as a comparison. The SDLY08 strain was isolated from our lab and its pathogenicity has been previously reported (Wang et al., 2010; Chen et al., 2015). The remaining 20 chickens were challenged with an equal volume of PBS, serving as a negative control group. Importantly, all chickens were immunized with an AIV-H9 inactivated oil emulsion vaccine. All chickens were monitored daily for the occurrence of clinical signs, and the growth or death of chickens was evaluated daily.

### 2.7 Testing indices of animal experiments

To evaluate the effects of viral infection on growth retardation and immunosuppression, individual body weights (BWs) were measured at the age of 10, 11, 12, 13, 14, and 15 weeks in all experimental groups. At 15 weeks of age, six chickens from each group were randomly chosen and euthanized, and the thymus and bursa of Fabricius were collected. Concomitantly, after weighing the organs, the immune organ indices of the thymus and bursa of Fabricius were calculated as organ weight according to the formula (wet weight, mg)/BW (g) × 100%.

In addition, heparin blood samples were collected from all chickens at the age of 10, 11, 12, 13, 14, and 15 weeks for DNA extraction to determine the viremia positive rate of CAV according to published real-time PCR methods (Ren et al., 2016). Moreover, the red blood count (RBC) was analyzed using a PE-6800vet automatic Hematology Analyzer (Prokan, Shenzhen, China). Sera from all chickens were collected at the age of 10, 11, 12, 13, 14, and 15 weeks for hemagglutination inhibition testing to determine the concentration of AIV-H9 antibodies. Finally, the CAV specific antibody titer was determined by enzyme-linked immunosorbent assay (IDEXX, Westbrook, ME, USA).

### 2.8 Statistical analysis

Statistical analysis was performed using the SPSS statistical software package for Windows, version 13.0 (SPSS Inc., Chicago, IL, USA). Differences between groups were examined for statistical significance using the Duncan’s multiple-range test. A p-value less than 0.05 was considered statistically significant.

## 3 Results

### 3.1 Viral isolation, propagation, and titration

We performed PCR analysis on bone marrow samples from two-month-old chickens exhibiting clinical signs of severe anemia in a large-scale layer farm in the Shandong Province, China. We found that all tested samples were positive for CAV. Of note, we did not detect any other pathogen, including ALV, AIV, NDV, REV, or FAdV, in these samples.

**Figure 1:**
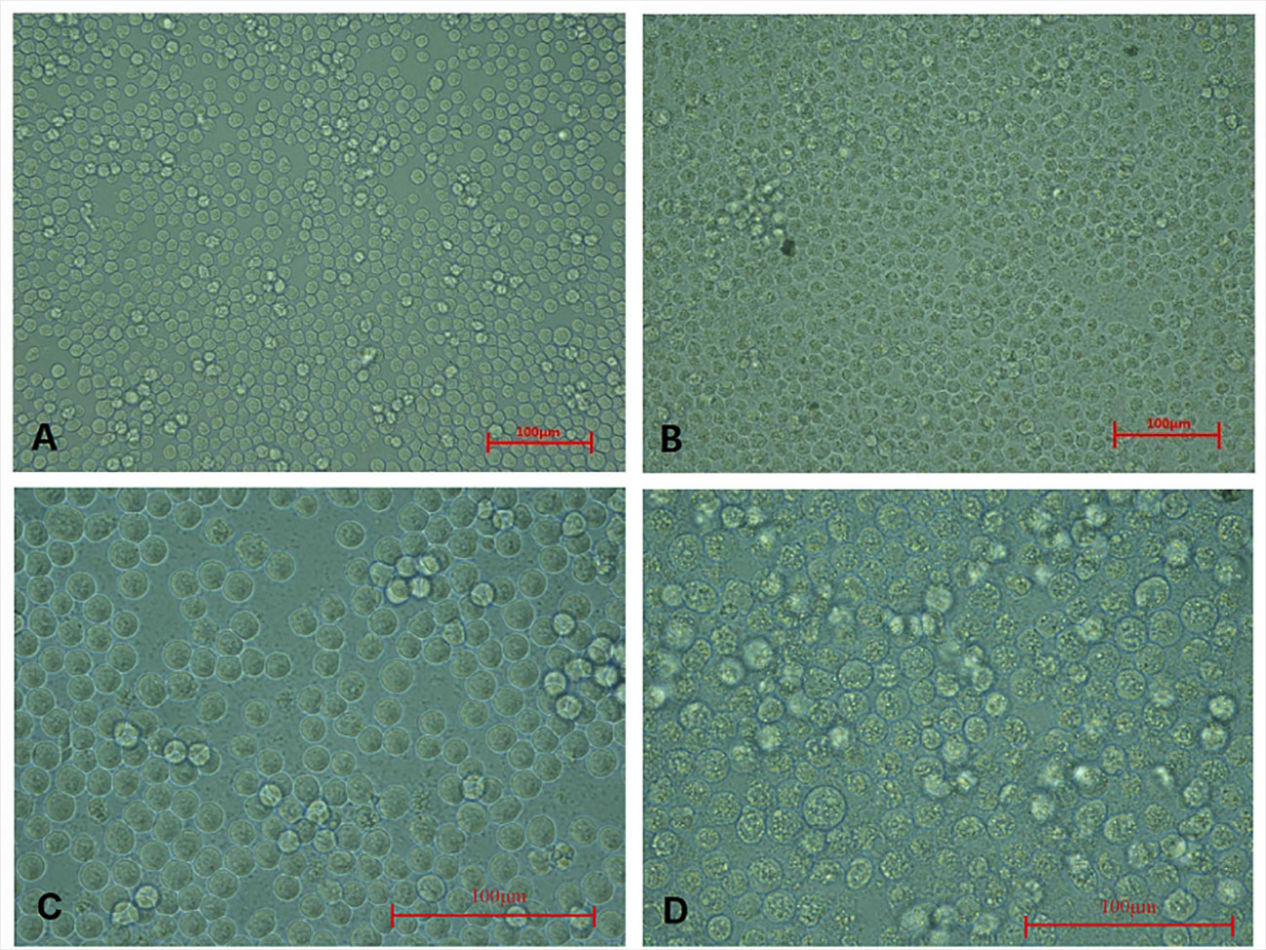
Cytopathic effect of CAV isolate on MSB1 cells. **(A)** Normal MSB1 cells (200×). **(B)** CPE of MSB1 cells infected with CAV (200×). **(C)** Normal MSB1 cells (400×). **(D)** CPE of MSB1 cells infected with CAV (400×).

**Figure 2:**
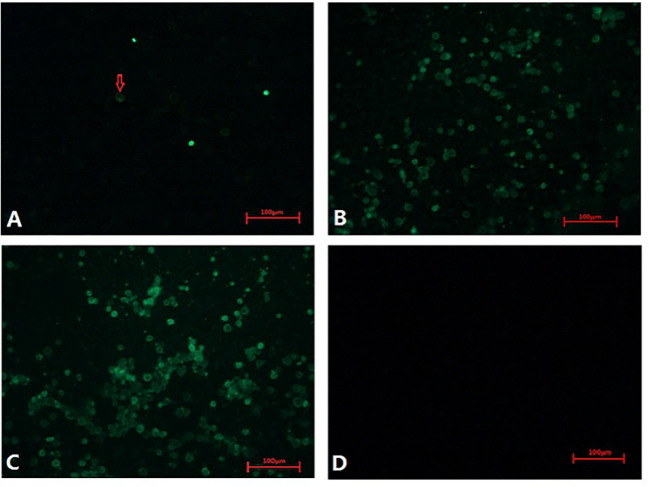
Infection of CAV isolate on MSB1 cells at different times. **(A)** 12 hours post-infection(200×). **(B)** 36 hours post-infection(200×). **(C)** 48 hours post-infection(200×). **(D)** Negative control(200×).

These CAV-positive samples were inoculated on MSB1 cells, which exhibited visible cytopathic effects (CPE), such as swelling followed by apoptosis, Normal MSB1 cells were shown in **Figures 1A, C** as negative control. As shown in **Figures 1B, D**, we observed the occurrence of typical apoptotic lesions of MSB1 cells at the third day after infection. This result demonstrated the successful propagation of the CAV strain in MSB1 cells. We named the novel CAV strain SD15. We also confirmed the propagation of CAV in MSB1 cells by IFA staining using mouse anti-CAV-VP3 monospecific serum (**Figure 2**). We performed IFA to observe the infection status of cells at different times. We accordingly observed an increasing number of infected cells at 12, 36, and 48 h post-infection (**Figures 2A-C**; negative control is shown in **Figure 2D**). We also detected that intranuclear inclusions appeared in some cells at 12 h post-infection. We determined that approximately half of cells were infected with CAV at 48 h post-infection.

We then injected purified CAV isolated from MSB1 cells in embryonating chicken eggs to measure the viral titer. We found that the average viral titer was 10^6^ EID_50_/mL.

### 3.2 Sequencing and phylogenetic analysis

**Figure 3:**
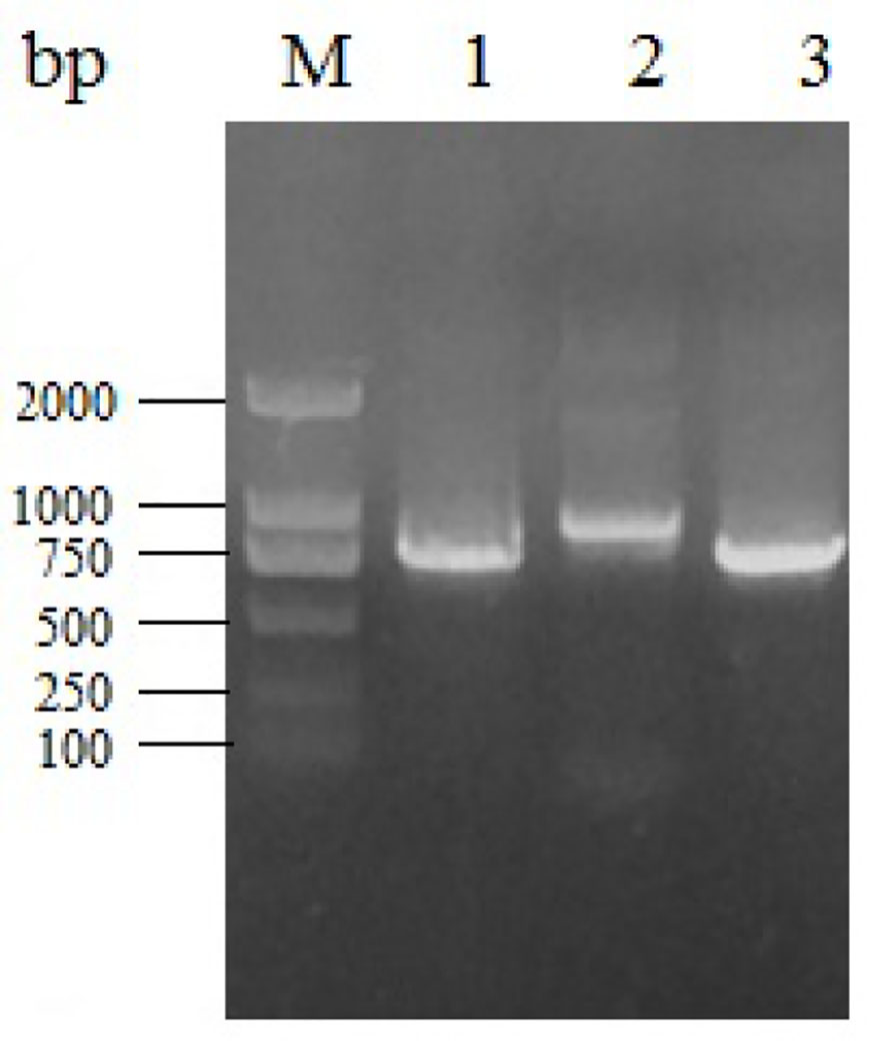
Electrophoresis of different fragments of CAV genome. M: Marker DL2000; 1, 2, 3: gene fragment of CAV-1, 2, 3.

We amplified the whole genome of the isolated CAV strain using three sets of primers (**Figure 3**). After thrice repeated sequencing, we did not detect any base mutations in the SD15 genome between the two sequenced samples (original bone marrow and cell supernatant). The complete genome sequence of CAV strain SD15 was submitted to GenBank, under the accession number KX811526. The length of the whole genome of SD15 was 2298 bp. The complete VP1, VP2, and VP3 gene sequences of SD15 were 1350, 651, and 366 nucleotides in length, respectively. We compared the whole genome sequence of SD15 to that of 33 other strains isolated from different areas and times (**Table 1**). Comparative analyses showed that strain SD15 shared the highest sequence identity at the nucleotide level (98.9%) with strain isolate 18 (accession No. KJ728827) isolated in Taiwan, whereas it exhibited the least (93.9%) with strain NC001427 (accession No. NC001427) isolated from the USA.

**Figure 4:**
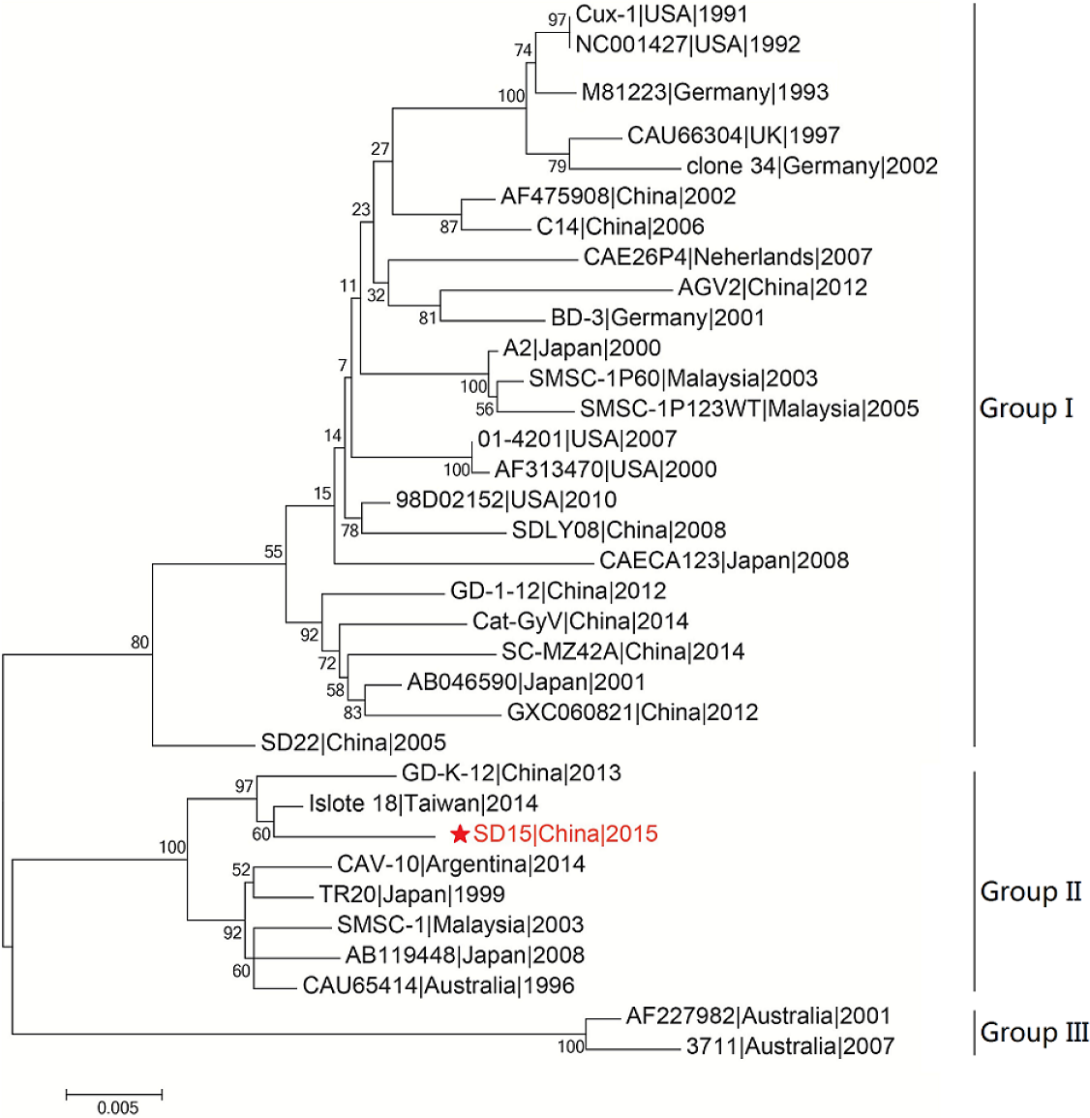
Phylogenetic analysis of CAV strain SD15 based on whole-genome. The sequence from the present study is named SD15 and is shown with a red star. GenBank sequences were given the strains name followed by country name and time. The three major groups were identified as Group I, Group II, and Group III. The whole sequences were analyzed by using MEGA5.1 sofware with neighbor-joining (NJ) phylogenetic tree methods together with the novel sequence. Each tree was produced using a consensus of 1000 bootstrap replicates.

We then performed phylogenetic analysis based on the comparison of the whole genome nucleotide sequence using the sequence of the novel CAV strain SD15 and the full-length genome sequences of 33 other strains (**Figure 4**). The Bayesian inference showed that CAV was classified into three major groups, 1–3, as supported by the topology and high posterior probability (generally >0.80) (**Figure 4**). In addition, we observed that 34 CAV sequences were scattered across different branches, without clear spatio-temporal distribution characteristics. Interestingly, we determined that the SD15 strain belonged in group II and was distributed on the same group with the isolate 18 strain isolated in Taiwan. These findings demonstrated the close phylogenetic relationship between the SD15 and isolate 18 strains.

### 3.3 Nucleotide sequence analysis of noncoding region of CAV genome

**Figure 5:**
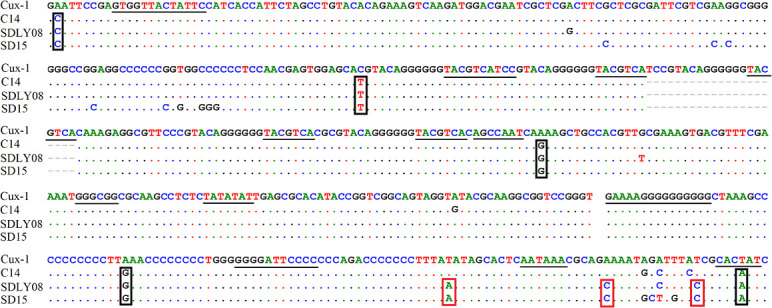
Nucleotide sequence analysis of noncoding region of CAV genome. The transcription factor binding sites are underlined. The black box represents the base mutations between Cux-1 and C14, SDLY08, and SD15. Red box represents the base mutations between low pathogenic strains (Cux-1 and C14) and the highly pathogenic strains (SDLY08, SD15).

We found that the noncoding region of CAV consisted of approximately 300 centrally distributed nucleotides, containing more than a dozen conserved sequences known to be related to replication and transcriptional regulation. We aimed to identify any differences in the noncoding region between different pathogenic strains. We compared the genome regulation-related motif of the noncoding region from reference strains with low (Cux-1 and C14 strains) and high (SDLY08 and SD15 strains) pathogenicity (**Figure 5**) (**Table 3**). We detected that the noncoding sequences, especially those of transcription factor binding sites (underlined), were conserved (nucleotide homology was 90.0–99.8%) across the genome of different CAV strains. Compared with the Cux-1 strain, the C14, SDLY08, and SD15 strains did not contain the CACTAT motif, lacked a CREB site, and carried five base mutations (marked with black box). We also observed that, compared with the low pathogenic strains (Cux-1 and C14), the highly pathogenic strains (SDLY08, SD15) carried three base mutations (marked with red box).

**Table 3:** Transcription factor-binding sequence elements.

Motif	Consensus Sequence	CAV Sequence
TATA box	G T A T A ( A / T ) A ( A / T )	T A T A T A T
CCAAT box	A G C C A A T	A G C C A A T
SP1 site	G G G C G G	G G G C G G
ATF site	A C G T C A	A C G T C A
CREB site	( T / G ) ( T / A ) C G T C A	T A C G T C A
Core element of the SV40 enhancer	G T G G(A / T ) ( A / T ) ( A / T )	G T G G T T A
Erythroid specific G-string	G G G G G G G G G G	G G G G G G G G G G
NFKB+H2TF1 sites	G G G G A T T C C C C	G G G G A T T C C C C
Lymphoid specific site	C T A T T C	C T A T T C
Pu Motif	9 purines	G A A A A G G G G G G G G G G
CACTAT	A T rich C A C T A T	C A C T A T
PEA-1 site	G G A A G T G A C T A A C	G A A A G T G A C T T T C
GT IIsite	G ( G / C ) T G T G G A A ( A / T ) G T	C G T T G C G A A A G T
MLTF	G G C C A C G T G A C C	T G C C A C T G T C G A
CACCC site:	C A C C C	CAGCC CATCC
Poly (A) signal	A A T A A A	A A T A A A

### 3.4 Amino acid analysis of different coding regions of CAV genome

We determined that the complete VP1, VP2, and VP3 gene sequences of the CAV strain SD15 were 1350, 651, and 366 nucleotides in length, respectively, lacking any nucleotide insertions or deletions. We identified 16 mutations in strain SD15, two of which were unique amino acid mutations (F210S in VP1, and L25S in Vp3) that had not been previously reported.

**Figure 6:**
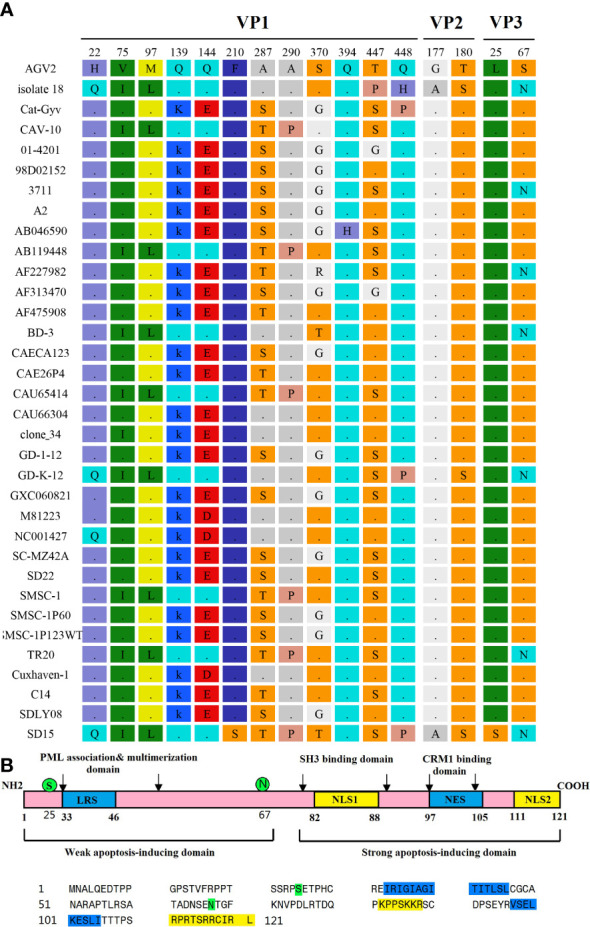
Amino acid mutations of CAV strain SD15. **(A)** Amino acid mutations in VP1, VP2, and VP3. **(B)** The primary structure of apoptin. The lower panel shows the sequence of apoptin (Strain SD15). Amino acids marked in blue or yellow represent the indicated domains in the upper panel. Amino acids marked in green represent mutations.

Previous studies reported that the VP1 protein exhibits the highest variability, with a hypervariable region located from residues 139 to 151, while amino acids 139 and 144 play vital roles in viral growth and spread in culture, as VP1 residues Q139 or Q144 or both have been associated with a decreased rate of spread (Renshaw et al., 1996). We found that, among the 34 CAV genome sequences, 10 strains had both Q139 and Q144 (including the SD15 strain), suggesting that these strains might have low rates of growth and spread *in vitro* (**Figure 6**). The remaining 21 CAVs contained residues K139 and E144, while three CAVs contained residues K139 and D144. In addition, the residue 394 of the VP1 protein has been reported to be a major genetic determinant of CAV virulence, with glutamine (Q) and histidine (H) representing high and low pathogenicity, respectively. Strain SD15 has glutamine at position 394 (**Figure 6A**), suggesting that it might be highly pathogenic. This was consistent with our following animal experiments.

Importantly, the CAV-induced apoptosis of cells has been attributed to VP3, a 14-kDa on-structural protein, which is therefore also referred to as apoptin (Noteborn et al., 1994; Maddika et al., 2006). The primary structure of apoptin is shown in **Figure 6B**. We determined that the SD15 strain carries two amino acid mutations in VP3: hydrophobic amino acid leucine mutated to hydrophilic amino acid serine at position 25 (L25S), and hydrophilic amino acid serine mutated to asparagine at position 67 (S67N). As shown in **Figure 6B**, these two mutations are positioned in the weak apoptosis-inducing domain and did not affect the bipartite nuclear localization sequence (NLS1 spanning amino acids 82–88, with NLS2 at residues 111–121) or putative nuclear export sequence (NES at residues 97–105).

### 3.5 Influence of infection of CAV on growth rate and immune organs

**Figure 7:**
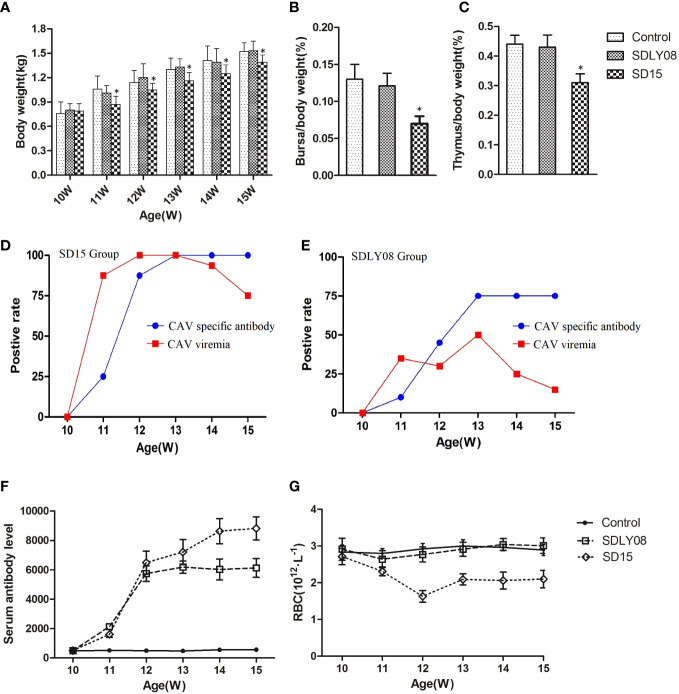
Animal experiment indices of infected chicks and uninfected controls. **(A)** The body weights of chickens in each group. **(B, C)** Average bursa and thymus were calculated as organ weight (g)/BW (g) x 100%. **(D, E)** The positive rate of CAV viremia and CAV specific antibody of blood samples in the SD15 and SDLY08 groups. **(F)** CAV specific antibody titers in each group. **(G)** The number of RBC in each groups. *Indicates significant difference (P< 0.05) based on Duncan’s multiple-range test.

We compared the body weights to show the effects of the novel SD15 and reference SDLY08 CAV strains on the growth rate of 10-week-old SPF chickens subjected to different treatments. Body weights are summarized in **Figure 7A**. We found that chickens inoculated with SD15 had significantly lower weights than those infected with SDLY08 (10.7%-19.2% in growth retardation) or the control group (10.4%-20.4% in growth retardation) at 11–15 weeks (7–35 dpi) (P < 0.05). However, we noticed that chickens infected with SDLY08 showed no significant growth retardation compared with the control group. These results demonstrated that the novel CAV strain SD15 seriously disturbed the growth of 10-week-old SPF chickens.

We euthanized six chickens from each group at the age of 15 weeks. Statistical analysis showed that the immune-organ-to-body weight ratio in the SD15 group was significantly lower than that in the SDLY08 and control groups (P < 0.05) at 35 dpi (**Figures 7B, C**). We then dissected the thymus and bursa of Fabricius of chicken from the three groups (**Figure S1**) and detected visible lesions only in the SD15-infected group (atrophied thymus with bleeding sites; shrunk bursa of Fabricius).

### 3.6 Influence of infection of CAV on viremia and specific antibody level

To assess the interaction of CAVs with the host, we determined the positive rate of viremia and specific antibody levels at 0, 7, 14, 21, 28, and 35 dpi. As shown in **Figure 7D**, the positive viremia rate reached 75%, while the positive rate of CAV specific antibody was 25% in the SD15-infected group at 7 dpi. We further found that the positive viremia rate reached 100% at 14 dpi. Interestingly, when the positive rate of CAV specific antibody reached 100% at 21 dpi, the positive viremia rate started to decline. However, we detected that the highest positive rate of viremia in the SDLY08-infected group was only 50% at 21 dpi. In addition, the highest positive rate of CAV specific antibody was 75% at 21 dpi (**Figure 7E**). We observed that, although the CAV specific antibody titers were continuously rising at 7–35 dpi in both CAV-infected groups (**Figure 7F**), the novel strain induced higher antibody titers. In addition, we did not detect any CAV or other virus specific antibody in the control group (not shown).

### 3.7 Influence of infection of CAV on red blood cells

We measured the number of red blood cells of the chickens at 0, 7, 14, 21, 28, and 35 dpi in both CAV-infected groups and the control group. As shown in **Figure 7G**, chickens exhibited severe anemia following infection with the CAV strain SD15, beginning at 7 dpi and continuing throughout the entire monitoring period. We found that the number of RBC dropped to the lowest point (173 × 10^12/^L) at 14 dpi, which was just 60% of that in the control group (291 × 10^12/^L). We observed that, compared with the control group, the number of RBC in the SDLY08 group only showed a slight drop at 7 and 14 dpi, and then recovered.

### 3.8 Influence of CAV infection on HI antibody titers against avian influenza virus subtype H9(AIV-H9)

As shown in **Table 4**, levels of HI titers against AIV-H9 appeared and were continuously increased in both infected and control groups after immunization of 10-week-old chickens with the inactivated AIV-H9 vaccine. However, we observed that the SD15-infected group exhibited significantly weaker humoral immune responses against the inactivated AIV-H9 vaccine. The HI titer in the SD15-infected group was significantly lower than that in the control group in the first 4 weeks post-infection (P < 0.05). Interestingly, we noticed that at 5 weeks post-infection, the level of H9 antibody in the SD15-infected group was significantly increased although still lower than that in the control group; however, no statistical difference was observed between the two groups (P > 0.05). Although we found that the levels of H9 antibody in the SDLY08-infected group were lower than those in the control group, the difference in the levels between the two groups was significant only at 14 dpi. These results indicated that SD15 caused stronger immunosuppression than SDLY08.

**Table 4:** HI antibody titers against AIV-H9 were measured at different days of age.

Group	HI antibody titters against AIV-H9 at different days of age(log2)					
	10w	11w	12w	13w	14w	15w
Control	0^a^	3.12^a^	4.11^a^	5.43^a^	6.28^a^	6.32^a^
SDLY08	0^a^	2.76^a^	3.23^b^	4.96^a^	5.89^a^	6.41^a^
SD15	0^a^	1.29^b^	2.14^c^	2.86^b^	4.43^b^	5.70^a^

Different lower case superscript letters indicate that the difference is statistically significant within a column (P<0.05) based on Duncan‘s multiple-range test.

## 4 Discussion

With the expansion of the poultry industry and its increasing stocking density in China, infectious diseases of chickens have gradually become more endemic, causing huge economic losses. Since its first isolation from the Heilongjiang Province in China (Cui et al., 1992), CAV has been widely distributed in China. Currently, no commercial CAV vaccine is available in China, hindering the effective control of CAV infections. The natural infection rate of CAV can be as high as 40–70%, with the seropositivity rate even reaching 100% in commercial chicken farms (Li et al., 2017). Despite the highly conservative CAV genome, the emergence of novel virulent strains has been reported under the immune selection pressure of high antibody titers. However, there is little in-depth research on the pathogenicity of CAV in chickens over 6 weeks of age.

In the present study, we successfully isolated a novel CAV strain (named SD15) from 2-month-old chickens and, to the best of our knowledge, this is the first study on the isolation of CAV strains from older chickens in China. Although CAV infection has been reported to be subclinical in older chickens (McNulty, 1991), infected chicken in the CAV outbreak chicken farm studied here showed severe anemia and pale bone marrow. Yuasa reported that chickens older than 2 weeks showed significant age-related resistance to the virus (Yuasa, 1988). So, it was important to know if the isolated virus (SD15) was of higher virulence. In view of this, we performed systematic analysis of the molecular characteristics and pathogenicity of the SD15 strain.

To explore the factors associated with the pathogenicity of CAV strains, we compared 34 strains isolated from different regions and time periods. The phylogenetic tree constructed using the 34 full-length CAV genomes clearly showed that strain SD15 was closely related to strain Isolate18 isolated in Taiwan (homology 98.9%). However, as no animal experiments were conducted using strain Isolate18, we could not compare the pathogenicity of these viral strains. In addition, phylogenetic analysis revealed that CAV genomes within the same genetic group lacked spatio-temporal distribution characteristics (**Figure 4**). Because there was no obvious correlation between genotyping and the time or region of viral isolation, it is possible for these viruses to have evolved independently in different regions. Consequently, to accurately trace the origin and explore the genetic evolution of CAV on the basis of existing evidence is not possible.

The noncoding region of viral genomes plays an important role in the replication and transcriptional regulation of the virus (Noteborn et al., 1991). In this study, the noncoding regions of four strains with different pathogenicity were compared. The reference strains Cux-1 and C14 are low-pathogenic, whereas strains SDLY08 and SD15 are high-pathogenic strains (**Figure 5**). In particular, strain Cux-1 is widely used as a vaccine strain. Strain comparison revealed three different bases between the low and highly pathogenic strains (marked with red box). Previous reports have found that the noncoding region sequences are highly conserved in the CAV genome (nucleotide homology of 90.0–99.8%). As these identified base mutations were highly consistent among strains with different pathogenicity, they have the potential to become markers of pathogenicity.

We also performed comparative amino acid analysis of 34 strains. Our results showed that SD15 had two unique amino acid mutations never reported previously: the hydrophobic phenylalanine (F) at the 210th position of the VP1 protein mutated to hydrophilic serine (S), and the hydrophobic leucine (L) at the 25th position of the VP3 protein mutated to hydrophobic serine (S). The variation of these two amino acids was related to the changes of hydrophilicity and hydrophobicity, which might alter the balance of protein structure and function. CAV strain SD15 has glutamine (Q) at the 394th position of the VP1 protein. This finding was in agreement with the study reporting that glutamine (Q) at the 394th position of the VP1 protein is a major genetic determinant of CAV virulence, representing high pathogenicity (Yamaguchi et al., 2001). In addition, Renshaw et al. reported that amino acids Q139 or Q144 in VP1 are associated with a decreased rate of spread (Renshaw et al., 1996). However, although strain SD15 has both Q139 and Q144 in VP1, its proliferation in MSB1 cells was not slow. This suggested that there were other factors that enhanced its proliferation ability in culture. Apoptin is the product of the CAV VP3 gene that mainly induces the apoptosis of chicken lymphocytes, and is thus associated with pathogenicity. Interestingly, we identified two amino acid mutations in this region (**Figure 6B**): hydrophobic leucine to hydrophilic serine (L25S) and hydrophilic serine to hydrophilic asparagine (S67N). Of note, these novel substitutions were spotted only in the weak apoptosis-inducing region and had no effect on the recognition sequences NES and NLS, which drive the shuttling of apoptin in and out of the nucleus (Danen-Van Oorschot et al., 2003; Kuusisto et al., 2008). We speculated that the mutations of these amino acid sites led to the altered virulence of CAV strain SD15. However, further investigation is required to support this notion.

Previous studies have reported a lack of typical clinical symptoms in 4- and 6-week-old chickens after infection with CAV (Haridy et al., 2012). We accordingly showed that strain SD15 was highly pathogenic to 10-week-old chickens. The clinical symptoms induced by SD15 were first reported in chickens older than 6 weeks. Although the reference strain SDLY08 was fatal to 1-d-old chickens, and caused clinical symptoms to 3-week-old chickens (Chen et al., 2015), it showed weak pathogenicity to 10-week-old chickens. Interestingly, no significant effect was observed in 10-week-old chickens in terms of growth rate, immune organ index, or immune function in the SDLY08-infected group, suggesting that the pathogenicity of the novel strain SD15 was higher than that of the reference strain SDLY08. Although strain SD15 caused clinical symptoms in older SPF chickens, obvious age resistance was also observed. Whereas it was deadly to young chickens, strain SD15 did not cause death in 10-week-old SPF chickens. In addition, with the increase in the levels of CAV specific antibody titers, the positive viremia rate started to decline at 21 dpi. These findings indicated that the health of chickens tended to recuperate. Nonetheless, our results demonstrated that the positive viremia rate was still 75% 5 weeks after infection (15–week-old chicken), with the levels of CAV specific antibody titers arising at 7 dpi (positive rate: 25%, 8/20) and gradually increasing throughout the entire monitoring period. The higher positive viremia rate and level of antibody titer indicated persistent infection. In view of the vertical transmission of the virus, the persistent infection of older chickens is worth our attention. It is necessary to emphasize the harmfulness of CAV infection in 10-week-old chickens, as the virus can spread vertically, posing a great threat to the next generation of chickens.

As no CAV vaccine is available in China, natural transmission of CAV provides an opportunity for CAV-infected breeding chickens to produce antibodies before laying eggs, the sufficiently high antibody titers of which might also protect some offspring. However, epidemiological investigations have fully demonstrated that there are some uncertainties about the natural transmission of the pathogen in commercial chicken farms in China. The wide spread of CAV has suggested that natural transmission failed to control the disease, raising important questions in the chicken industry. In this study, we found that the novel CAV strain still had strong pathogenicity to older chickens and might also spread to chicks, suggesting that there is a great risk in relying on natural (or artificial) infection to provide protection to chicks.

In conclusion, this study was the first to report the isolation of a highly pathogenic CAV strain from 2-month-old chickens in China. Our genomic analysis highlighted some genetic characteristics of the highly pathogenic novel CAV strain that distinguish it from other CAV strains. Our study provided a better understanding of the critical factors determining the pathogenicity of CAV strains and can raise awareness of the molecular characterization and genetic diversity of CAV isolates still circulating in China. Finally, the high pathogenicity of the novel strain was verified on 10-week-old SPF chickens. These data demonstrated that the pathogenicity of epidemic CAV strains might increase and that CAV has the potential ability to break age resistance. Overall, these findings warrant additional efforts to explore CAV genomics and epidemiology, which might eventually lead to better control of CAV infections.

## Data availability statement

The original contributions presented in the study are included in the article/**Supplementary Material**. Further inquiries can be directed to the corresponding authors.

## Ethics statement

The animal study was reviewed and approved by Institutional Animal Care and Use Committee of Shandong Academy of Agricultural Sciences.

## Author contributions

LF performed the experiments, analyzed the data, and drafted the manuscript; PZ and LQ supervised the project and edited the manuscript; YH and HJ conducted part of the experiments; YW analyzed part of the data; and ZC provided important suggestions. All authors have read and agreed to the published version of the manuscript.

## Supplementary material

Supplementary Figure 1 The visible lesions of the thymus and bursa of Fabricius of the three groups.
