# Supplementary figures and images for: Molecular characterization and pathogenicity study of a highly pathogenic strain of chicken anemia virus that emerged in China
Source: Front Cell Infect Microbiol. 2023 May 22;13:1171622. doi: 10.3389/fcimb.2023.1171622 (PMC10240067; doi:10.3389/fcimb.2023.1171622)

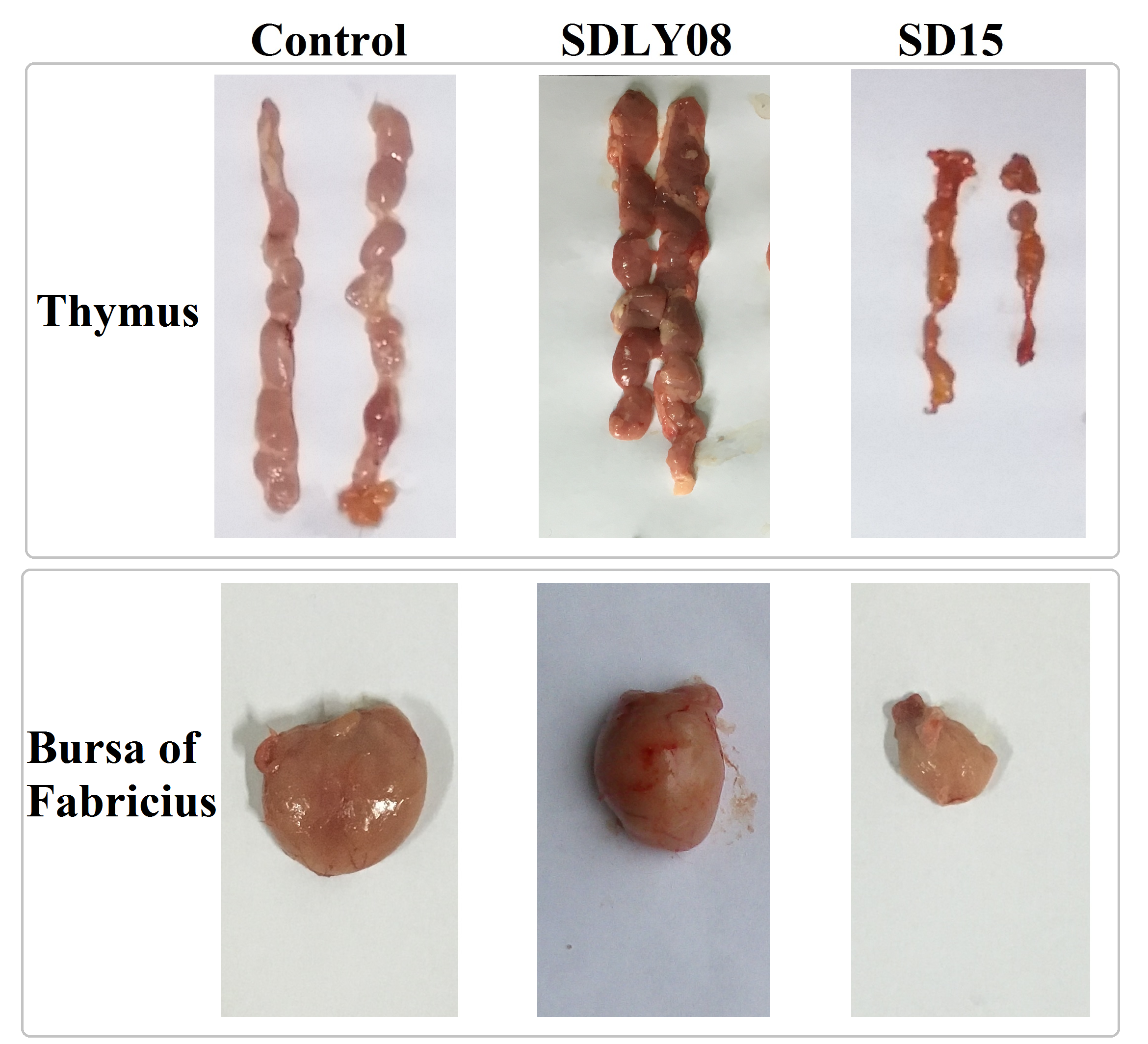

Supplement: Supplementary Figure 1 — The visible lesions of the thymus and bursa of Fabricius of the three groups. [file Image_1.tif]
